# A novel biliary stent coated with silver nanoparticles prolongs the unobstructed period and survival via anti-bacterial activity

**DOI:** 10.1038/srep21714

**Published:** 2016-02-17

**Authors:** Fuchun Yang, Zhigang Ren, Qinming Chai, Guangying Cui, Li Jiang, Hanjian Chen, Zhiying Feng, Xinhua Chen, Jian Ji, Lin Zhou, Weilin Wang, Shusen Zheng

**Affiliations:** 1 Key Laboratory of Combined Multi-organ Transplantation, Ministry of Public Health, First Affiliated Hospital, School of Medicine, Zhejiang University, Hangzhou 310003, China; 2 Department of Hepatobiliary and Pancreatic Surgery, First Affiliated Hospital, School of Medicine, Zhejiang University, Hangzhou 310003, China; 3 Collaborative Innovation Center for Diagnosis and Treatment of Infectious Diseases, Zhejiang University, Hangzhou 310003, China; 4 Department of Disinfection Supply Room, First Affiliated Hospital, School of Medicine, Zhejiang University, Hangzhou 310003, China; 5 State Key Laboratory for Diagnosis and Treatment of Infectious Diseases, First Affiliated Hospital, School of Medicine, Zhejiang University, Hangzhou 310003, China; 6 Department of Anesthesiology, First Affiliated Hospital, School of Medicine, Zhejiang University, Hangzhou 310003, China; 7 Department of Polymer Science and Engineering, Key Laboratory of Macromolecule Synthesis and Functionalization of the Ministry of Education, Zhejiang University, Hangzhou 310027, China

## Abstract

Symptomatic biliary stricture causes life-threatening complications, such as jaundice, recurrent cholangitis and secondary biliary cirrhosis. Fully covered self-expanding metal stents (FCSEMSs) are gaining acceptance for treatments of benign biliary stricture and palliative management of malignant biliary obstructions. However, the high rate of FCSEMS obstruction limits their clinic use. In this study, we developed a novel biliary stent coated with silver nanoparticles (AgNPs) and investigated its efficacy both *in vitro* and *in vivo*. We first identified properties of the AgNP complex using ultraviolet detection. The AgNP complex was stable without AgNP agglomeration, and Ag abundance was correspondingly increased with an increased bilayer number. The AgNP biliary stent demonstrated good performance in the spin-assembly method based on topographic observation. The AgNP biliary stent also exhibited a long-term anti-coagulation effect and a slow process of Ag^+^ release. *In vitro* anti-bacteria experiments indicated that the AgNP biliary stent exhibited high-efficiency anti-bacterial activity for both short- and long-term periods. Importantly, application of the AgNP biliary stent significantly prolonged the unobstructed period of the biliary system and improved survival in preclinical studies as a result of its anti-microbial activity and decreased granular tissue formation on the surface of the anastomotic biliary, providing a novel and effective treatment strategy for symptomatic biliary strictures.

Symptomatic biliary strictures can lead to a series of adverse outcomes, such as chronic cholestasis, jaundice, recurrent cholangitis and secondary biliary cirrhosis, and these complications can be serious, irreversible, and life threatening^1^. Biliary strictures can be caused by a variety of factors, including post-operation biliary fistula, primary sclerosing cholangitis^2^, pancreatitis^3^,^4^ and biliary complications after liver transplantation^5^, as well as malignant biliary obstructions. In total, 10–30% of patients with advanced chronic pancreatitis experience a symptomatic biliary stricture^6^, 4–9% of patients following orthotopic liver transplantation (OLT) develop anastomotic strictures^7^, and 0.3–0.7% of patients after laparoscopic cholecystectomy may suffer from a major bile duct injury, leading to post-operative strictures^6^. Thus, it is essential to provide prompt, effective and durable treatment of strictures.

Fully covered self-expanding metal stents (FCSEMSs) have been recognized as an optimal therapy for benign biliary strictures and palliative management of malignant biliary obstructions^1^,^8^,^9^,^10^. However, the long-term unobstructed rate of FCSEMSs in clinical usage remains limited. In benign biliary strictures, the average unobstructed period is approximately 6–9 months, whereas the average period for malignant biliary obstructions is 4–8 months^11^. The formation of bacterial biofilm is the main cause for the re-stricturing of biliary stents in clinical applications^12^,^13^, and bacterial infection is the leading factor for bacterial biofilm formation^13^,^14^. Therefore, development of a novel biliary stent with anti-microbial functionality is urgently needed for clinical applications.

Recent applications of nanotechnology in translational medicine require materials and devices designed to interact with the body on subcellular (i.e., molecular) scales with a high degree of specificity^15^ that may be potentially translated into targeted cellular and tissue-specific clinical applications to achieve maximal therapeutic efficacy with minimal side effects. Because of a high surface-to-volume ratio, nanoparticles have become a well-recognized, effective antimicrobial substance^16^,^17^,^18^. In this study, we developed a novel biliary stent coated with silver nanoparticles (AgNPs) and investigated its function *in vitro* and *in vivo*. We found that the AgNP biliary stent possessed a strong advantage and could resist a variety of bacterial infections *in vivo* and *in vitro*, significantly contributing to the long-term unobstructed status of the biliary system. Importantly, the application of the AgNP biliary stent significantly prolonged the unobstructed period of the biliary system and improved survival time in preclinical studies.

## Materials and Methods

### Preparation of chitosan nanosilver solution

A total of 0.0849 g silver nitrate was gently added to a 50 ml chitosan solution (pH = 3.8), and then both were well mixed as a 10 mmol/l Ag^+^ chitosan solution. Sixty milligrams of sodium borohydride was dissolved in 20 ml of distilled deionized water (ddH_2_O), and then the 5 ml mixture was added to the above Ag^+^ chitosan solution at a speed of 0.01 ml per second and 30 min stirring. The above solution was treated with a microfiltration membrane to discard larger particles and prevent particle aggregation. The obtained chitosan nanosilver solution was stored in a 4 °C refrigerator; the quality of the AgNPs was detected with ultraviolet detection at different time points, as previously described^19^.

### Preparation of Ag-loaded heparin/chitosan multilayer film coats

To coat a multilayer film on both the external and inner layers of the polyester used in a plastic biliary stent, the polyester was treated with small-molecule hexamethylenediamine to obtain positive electricity^20^. Multilayer film coats can be formed according to two methods: dip assembly and spin assembly^21^. The dip-assembly method was performed with the following steps. The polyester with positive electricity was dipped into a 1 mg/ml heparin solution for 15 min and was washed three times in ddH_2_O. Then, the polyester was dipped into a 5 mg/ml chitosan nanosilver solution for 15 min and washed three times in ddH_2_O. The above process was cycled 18 times, and 18 layers of film coats were obtained. The spin-assembly method was performed with the following steps. One milligram per millilitre of heparin solution (pH = 3.8) with a negative charge was dropped into the polyester with positive electricity and spin coated once. Then, the ddH_2_O was dropped into the polyester and spin coated once; this step was repeated. Finally, 5 mg/ml of chitosan nanosilver solution (pH = 3.8) with a positive charge was dropped into the polyester and spin coated once; the spin was at 4000 revolutions per second for 30 seconds. The above process was cycled 18 times, and 18 layers of film coats were obtained.

### Property characterization of Ag-loaded heparin/chitosan multilayer film coats

All multilayer film coat samples were vacuum dried overnight. The contact angles were measured with the circle fitting method on a JY-82-type contact-angle metre to track the assembly process of the multilayer film coats. A total of 18 layers of film coats were created with the spin-assembly method; the odd-numbered layers were dropped with heparin, and the even number layers were dropped with Ag-chitosan. The ultraviolet-visible spectrum was obtained on the ultraviolet and visible spectrophotometer (Cary 100 BIO, USA) to characterize the AgNPs and track the assembly process. The Ag-loaded multilayer film coats were dipped into hydrofluoric acid for 3–5 seconds and then dropped onto the copper wire mesh. The surface topography of the multilayer film coats was observed with an atomic force microscope (AFM) (SPI 3800, Seiko Instruments, Japan) and scanning electron microscopy (SEM) (FESEM SIRION100, FEI Incorporated, Holland) as previously described^22^.

### Detection of re-calcification clotting time

One millilitre of human plasma (preheated at 37 °C to discard Ca^2+^) was dropped onto the surface of the polyester, and then 1 ml of 0.025 mol/l CaCl_2_ solution was added. Simultaneously, a small stainless steel hook was stretched into the solution and mixed gently. The formation of fibrin was evaluated, and the time that the filament was emerged was recorded. The time is the re-calcification clotting time. Each sample was measured six times. In addition, Ag-loaded heparin/chitosan multilayer film coats were stored in a 4 °C refrigerator for 14 days and then used for measurement of the re-calcification clotting time, as previously reported^23^.

### Measurement of Ag^+^ concentration in the AgNP biliary stent at different time points

A Ag^+^ release experiment was conducted. The AgNP biliary stent was placed into 30 ml of PBS, and the new PBS solution was changed every 24 hours. At different time points, Ag^+^ concentrations in the PBS solution were measured and recorded using inductively coupled plasma mass spectrometry (ICP-MS). Each experiment was repeated at least three times.

### Anti-bacteria experiment of Ag heparin/chitosan multilayer film coats *in vitro*

#### Escherichia Coli (E. coli) detection

The polyester was placed in the weighing bottle. One millilitre of phosphate buffer solution (PBS) containing *Escherichia coli* at a concentration of 5 × 10^4^ cells/ml was dropped onto the polyester, and then the polyester was cultured in a 37 °C constant-temperature incubator. After 0, 1, 2, 4 and 8 hours, 0.1 ml of bacterial solution was removed, diluted and mixed with 0.9 ml of PBS. Then, 0.2 ml of diluted bacterial solution was homogeneously coated on the solid medium through the spread-plate method. After 24 hours of cultivation in a 37 °C constant temperature incubator, the bacterial colony was counted and the bacterial survival ratio was calculated. The polyester without multilayer film coats served as the control, and the polyester with multilayer film coats served as the AgNP group. Additionally, the polyester with multilayer film coats that was stored for 1 month in a 4 °C refrigerator was observed as the AgNP-1M group. Each experiment was repeated at least three times.

#### Other bacteria detection

According to the above methods, other bacteria, including *Staphylococcus aureus* (*Staph. aureus*), *Enterococcus* and *Pseudomonas aeruginosa* (*P. aeruginosa*), were stained and detected using the corresponding commercially bacteria chromogenic medium. The anti-bacterial effects of the AgNP group and control group were observed and recorded under a light microscope. The bacterial number and intensity were calculated as the integral optical density (IOD) per high-power field (HPF) using Image-Pro Plus (IPP) software. Each experiment was repeated at least three times.

### Animal care

A total of sixty-four Bama experimental miniature pigs (30–40 kg) were maintained in the Center of Animal Laboratory of Zhejiang University. Each pig was housed in a singular standard cage in an air-conditioned room (20–25 °C) with a 12 h light/12 h dark cycle, and a standard laboratory diet and water were provided. All animals received appropriate humane care from certified professional staff. Animal treatment protocols were approved by the Animal Care and Use Committee of Zhejiang University (reference number: 2015-194). The methods were carried out in accordance with the approved guidelines.

### Animal study design

The sixty-four pigs were randomly divided into two groups: the AgNP group (n = 32) and the control (n = 32) group. In the AgNP group, the novel AgNP biliary stents were inserted into the common bile ducts in all of the pigs. Teflon biliary plastic stents (Cook Medical Co., LTD, USA) served as the control group. For each group, eight pigs were randomly selected to observe the percentage of biliary obstruction and survival function, and B ultrasound was used to monitor the position and obstruction of the biliary stent once each month. The other 24 pigs were randomly divided into four time points for sample collection and bacterial infection detection (n = 6 at each time point).

### Surgical process for biliary stent implantation

**Figure 1:**
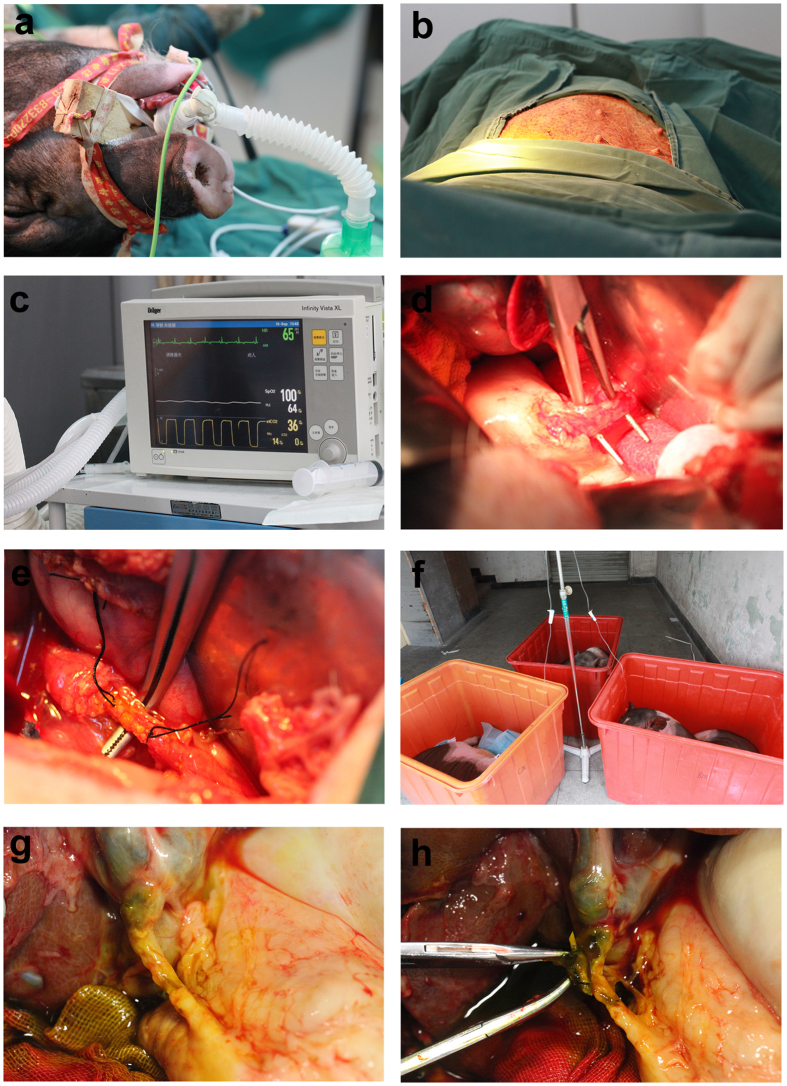
Surgery process of biliary stent implantation and collection in pigs. (**a**) Animals were managed with general anaesthesia and mechanical ventilation. (**b**) A neuromuscular blockade was administered to ensure complete paralysis. **(c**) Vital signs were monitored in real time using vital sign monitors. (**d**) Common bile ducts were gently dissociated and cut out, and approximately 5 cm-long biliary stents were implanted into the biliary ducts. (**e**) To suture the biliary duct, 5–0 absorbable suture material was used. (**f**) Animals were fasted from food and water for 12 hours, and appropriate antibiotics were administered to prevent infections. (**g**) The common bile duct with a biliary stent was gently dissociated. (**h**) The biliary stent was collected.

Animals were managed with general anaesthesia and mechanical ventilation (Fig. 1a), and a neuromuscular blockade was administered to ensure complete paralysis (Fig. 1b). Vital signs were monitored in real time by vital sign monitors (Infinity Vista XL) (Fig. 1c). Approximately 1 cm of the common bile ducts was gently dissociated and cut out (Fig. 1d), and biliary stents of approximately 5 cm in length were implanted into the biliary ducts. To suture the biliary duct, 5–0 absorbable suture material was used (Fig. 1e). One end of the biliary stent was inserted into the duodenum, and its location was confirmed by the operator. Animals had not received food or water for 12 hours, and appropriate antibiotics were administered to prevent infections (Fig. 1e). No animals presented complications.

### Sample collection

For 24 pigs in each group, blood samples were collected serially via an ear vein to monitor blood, liver function and cytokine alterations 4, 12, 24 and 48 weeks after biliary stent implantation. At each time point for each group, six pigs were managed with general anaesthesia. The common bile duct with a biliary stent was gently dissociated (Fig. 1g), and the biliary stent was obtained (Fig. 1h). Additionally, the bile was collected to conduct bacterial culture and identification. Anastomosis of the bile duct was performed to perform haematoxylin and eosin (H&E) staining and Masson’s trichrome staining.

### Liver function test and pathology staining

A plasma liver function test was conducted using an automatic biochemical analyser (Hitachi 7600, Tokyo, Japan) as previously described^24^. Bile duct pathology was observed with H&E staining following our previous protocol^25^,^26^. Masson’s trichrome staining for connective tissue was performed using a kit (#K037, Poly Scientific, Bay Shore, NY). The stained sections were analysed blindly under light microscopy by a pathologist.

### Statistical analysis

The data are presented as the mean ± standard error (SEM). For parametric data, statistical significance between groups was compared using Students’ T-Test. The survival distribution function was evaluated with the Kaplan-Meier survival curve. Statistical analyses were performed with the software package SPSS for Windows (version 19.0; SPSS, Inc., Chicago, IL, USA). A p-value of less than 0.05 was considered statistically significant.

## Results

### Property characterization of Ag-loaded heparin/chitosan multilayer film coats

**Figure 2:**
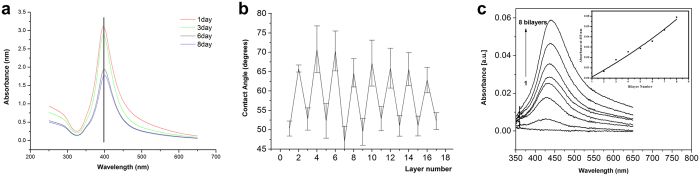
Property characterization of Ag-loaded heparin/chitosan multilayer film coats. (**a**) The quality of AgNPs was characterized by ultraviolet detection at different time points. The peak position of the wavelength of AgNPs was stable at 396 nm during the initial 8 days. (**b**) Contact angle of chitosan-Ag0/heparin multilayer films versus the layer number. Heparin and chitosan almost exhibited a strict alternate layer-upon-layer arrangement. (**c**) UV-visible spectra of chitosan-Ag0/heparin multilayer films versus the bilayer number. All multilayer film coats at different bilayers presented a maximum absorption at 433 nm wavelength. The maximum absorption presented a linear increase with the increased number of bilayers during the assembly process.

The quality of AgNPs was characterized using ultraviolet detection at different time points. The peak position of the wavelength of AgNPs was stable at 396 nm during the initial 8 days (Fig. 2a), which suggested that the obtained Ag-loaded chitosan complex was stable without AgNP agglomeration.

The contact angles were measured to track the assembly process of multilayer film coats. The result indicated that heparin and chitosan almost presented a strict alternate layer-upon-layer arrangement (Fig. 2b). This configuration might occur because the outermost layer of the polyester could be completely coated, receiving only minimal reciprocal crossover from the second layer. Because of the increased hydrophilia of heparin versus chitosan, the odd layer containing more heparin presented a smaller contact angle than the even number layer containing more Ag-chitosan.

The ultraviolet-visible spectrum was detected to characterize AgNPs and track the assembly process. Because of the effective ultraviolet absorption of AgNPs at 433 nm, all multilayer film coats at different bilayers presented a maximum absorption at the 433 nm wavelength (Fig. 2c). The maximum absorption presented a linear increase with the increase of the bilayer during the assembly process, which suggested that AgNP-chitosan was assembled layer by layer. Along with an increased bilayer number, the abundance of Ag in the multilayer film was correspondingly increased, thereby increasing the absorption of the AgNPs.

### Topography and effect of AgNPs biliary stent

**Figure 3:**
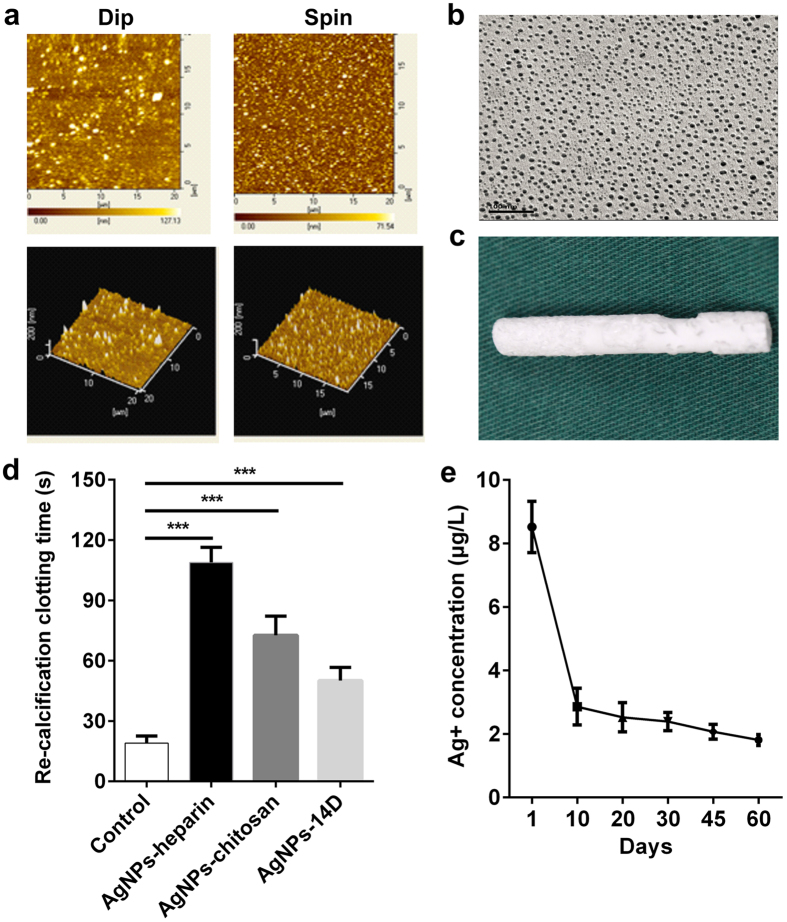
Topography and effect of the AgNP biliary stent. (**a**) The surface topography of the AgNP multilayer film coats from the dip-assembly and the spin-assembly methods, respectively, was observed with atomic force microscope (AFM). (**b**) The surface topography of AgNP multilayer film coats from the spin-assembly methods was observed with scanning electron microscopy (SEM). (**c**) The macroscopic morphology of the AgNP biliary stent is shown. (**d**) The influence of the AgNP biliary stent on coagulation function was evaluated based on the detection of re-calcification clotting time. The blank polyester served as the control. AgNPs-heparin: heparin as the outermost layer of the polyester; the polyester with AgNPs-chitosan: chitosan as the outermost layer of the polyester. ***p < 0.001. (**e**) A Ag^+^ release experiment was conducted through measurements of Ag^+^ concentration at different time points using the inductively coupled plasma mass spectrometry (ICP-MS).

The surface topography of the AgNPs biliary stent was observed using AFM and SEM. We used the tapping mode of AFM to observe the surface topography of multilayer film coats from the dip-assembly and the spin-assembly methods. As shown in Fig. 3a, different assembly methods exerted a significant influence on the roughness of the multilayer film. The surface of the multilayer film from the spin-assembly method presented a low roughness (root mean square (RMS) = 14.73 nm) versus the dip-assembly method (RMS = 22.37 nm). Because of less crossover between layers during the spin-assembly process, a flatter-surfaced multilayer film was obtained with the spin-assembly method. Additionally, the assembly of 18 layer coats required only 30 min with the spin-assembly method but 330 min with the dip-assembly method; thus, the spin-assembly method presented higher quality and efficacy. Furthermore, we observed the distribution of AgNPs in multilayer film coats produced with the spin-assembly method under SEM and noted that almost all AgNPs presented a uniform distribution. The diameter of each AgNP ranged from 5 to 15 nm (Fig. 3b). The macroscopic morphology of the AgNP biliary stent is shown in Fig. 3c.

The influence of the AgNP biliary stent on coagulation functionality was evaluated based on the detection of re-calcification clotting time. The blank polyester served as the control. We found that the polyester with AgNPs-heparin (heparin as the outermost layer) presented a significantly increased re-calcification time (108.8 ± 3.1) versus the blank polyester (19.0 ± 1.5, p < 0.001) (Fig. 3d). The polyester with AgNPs-chitosan (chitosan as the outermost layer) also displayed a remarkably prolonged re-calcification time (72.7 ± 3.9) versus the control (p < 0.001). Importantly, after storage of the AgNP biliary stent for 14 days, it still presented a longer re-calcification time (50.2 ± 2.7) than the control (p < 0.001), suggesting that the AgNP biliary stent exerted a long-term anti-coagulation effect.

To investigate the effect duration of the AgNP biliary stent, a Ag^+^ release experiment was conducted through measurements of Ag^+^ concentration using ICP-MS. We found that the concentration of Ag^+^ release achieved the maximum (8.52 ± 0.81 μg/l) on the first day, was significantly decreased during the initial 10 days (2.86 ± 0.58 μg/l), and then gradually reduced after 20, 30, 45 and 60 days, corresponding to 2.53 ± 0.46, 2.39 ± 0.29, 2.07 ± 0.23 and 1.81 ± 0.17 μg/l, respectively (Fig. 3e). These data indicated that the Ag^+^ release in the AgNP biliary stent was an effective and slow process.

### Anti-bacterial activity of the AgNP biliary stent *in vitro*

Because of a high incidence of bacterial infection after biliary injury^27^, bacterial adherence and biliary sludge are prone to occur, inducing biliary stent occlusion; thus, we investigated the anti-bacterial activity of the AgNP biliary stent.

**Figure 4:**
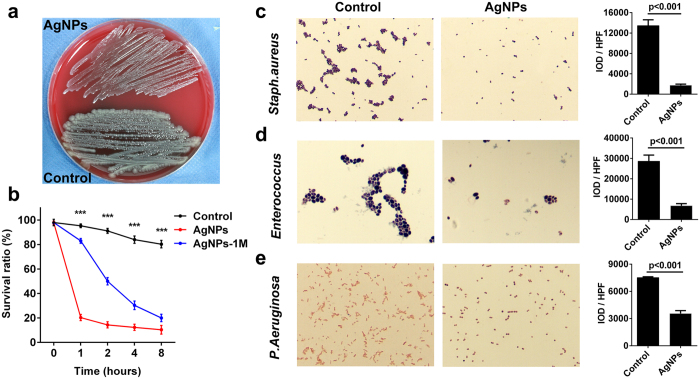
Anti-bacterial function of the AgNP biliary stent *in vitro.* (**a**) The colony number of *E. coli* was significantly decreased after 1 hour of co-culture of AgNPs and *E. coli* versus the control. (**b**) The survival ratios of *E. coli* were compared at different time points of AgNPs and *E. coli* co-culture versus the control. AgNPs-1M: AgNPs biliary stent storage for 1 month. (**c**) The colony number of *Staph. aureus* was compared in AgNPs and *E. coli* co-culture versus the control. Magnification: 400×. (**d**) The colony number of *Enterococcus* was compared in AgNPs and *E. coli* co-culture versus the control. Magnification: 400×. (**e**) The colony number of *P. aeruginosa* was compared in AgNPs and *E. coli* co-culture versus the control. Magnification: 400×. IOD: integral optical density; HPF: high-power fields. The bacterial number was determined with the spread plate method.

We first detected the anti-*E. coli* effect of the AgNP biliary stent. After 1 hour of the co-culture of AgNPs and *E. coli*, the colony number of *E. coli* was significantly decreased versus the control (Fig. 4a), and the survival ratio of *E. coli* was also remarkably reduced in the AgNP group versus the control (p < 0.001, Fig. 4b). After 2, 4 and 8 hours of the co-culture of AgNPs and *E. coli*, the survival ratio of *E. coli* presented a continuous decrease versus the control (all p < 0.001). Notably, after storage of the AgNP biliary stent for 1 month, the survival ratio of *E. coli* continuously declined versus the control under the co-culture of AgNPs and *E. coli* (all p < 0.001, Fig. 4b), which suggested that the AgNP biliary stent exhibited a high-efficiency anti-bacterial activity for both short- and long-term periods.

After biliary injury, the infected bacteria usually include *Staph. aureus*, *Enterococcus* and *P. aeruginosa*, in addition to *E. coli*. *Staph. aureus* is representative of gram-positive bacteria and can be identified using a commercially available *Staph. aureus* chromogenic medium. We found that the colony number of *Staph. aureus* was significantly decreased in the AgNP group versus the control (p < 0.001, Fig. 4c). In addition, the colony numbers of *Enterococcus* and *P. aeruginosa* were remarkably reduced in the AgNP group versus the control (both p < 0.001, Fig. 4d,e). These data suggested that the AgNPs biliary stent possessed a strong, broad-spectrum anti-bacterial function.

### AgNP biliary stent prolonged the unobstructed period and survival *in vivo*

**Figure 5:**
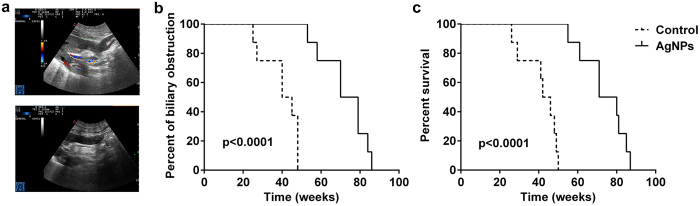
AgNP biliary stent prolonged the unobstructed period and survival *in vivo.* (**a**) Biliary dilatations in both the intrahepatic and extrahepatic bile duct (top) and biliary stent position in the common bile duct (below) at one month after the operation were observed with B ultrasound. (**b**) The percentage of biliary obstruction occurrence in the AgNP group and the control group were compared using a Kaplan-Meier survival curve. (**c**) The Kaplan-Meier survival curve demonstrated the survival function in sixteen pigs from the AgNPs group versus the control group.

To further investigate the effect of AgNPs biliary stents, we performed a preclinical study in sixty-four pigs. For each group, eight pigs were randomly selected in which to observe the percentage of biliary obstruction and survival function. One month after the operation, there were no biliary dilatations in either the intrahepatic or extrahepatic bile duct (Fig. 5a, top) and the biliary stent was in a proper position in the common bile duct (Fig. 5a, below), as shown by a B ultrasound. Commonly, biliary obstruction is characterized by various degrees of chills and high fever, notably yellow skin, and poor eating and activity. Our results indicated that the average time of biliary obstruction was significantly prolonged in the AgNP group (72.37 ± 4.23 weeks) versus the control group (40.13 ± 3.30 weeks, p < 0.0001, Fig. 5b), which suggested that the application of the AgNP biliary stent significantly delayed the occurrence of biliary obstruction versus the control. Correspondingly, the animals died approximately one to two weeks following biliary obstruction. The average survival time was 73.88 ± 4.06 weeks in eight pigs from the AgNP group, whereas the average survival time was only 41.38 ± 3.24 weeks in the control group. The Kaplan-Meier survival curve demonstrated that the survival function was remarkably increased in the AgNP group versus the control (p < 0.0001, Fig. 5c). These data indicated that the application of the AgNP biliary stent significantly prolonged the unobstructed period of the bile duct and improved survival time in the preclinical study.

### The AgNP biliary stent maintained a good biliary status *in vivo*

**Figure 6:**
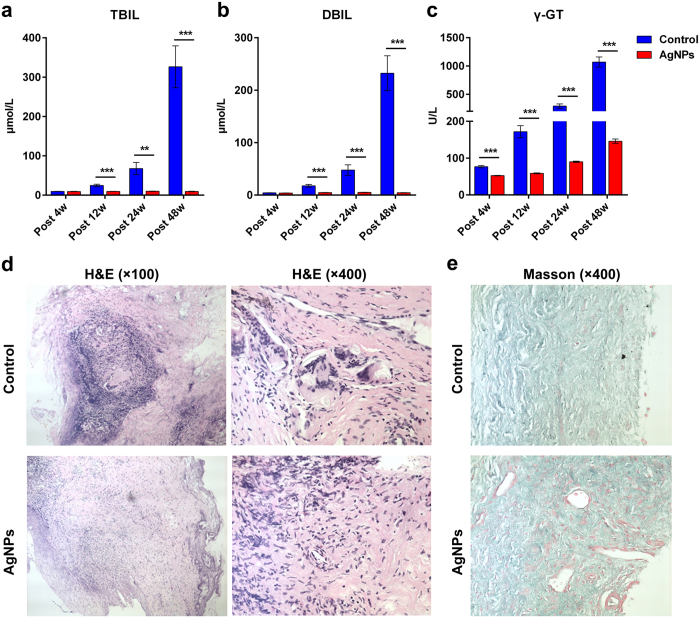
AgNP biliary stent maintained good biliary status *in vivo.* To monitor biliary status in real time, blood samples were collected serially via an ear vein at 4, 12, 24 and 48 weeks after biliary stent implantation to test liver function in the other twenty-four pigs for each group. Plasma levels of (**a**) total bilirubin (TBIL) and (**b**) direct bilirubin (DBIL) and (**c**) γ-L-glutamyl dipeptide (γ-GT) were compared between the AgNP group and the control group at different time points post-operation. **p < 0.01; ***p < 0.001. (**d**) The histology of bile duct anastomosis in the AgNP group and control group was observed by haematoxylin and eosin (H&E) staining. Magnification: 100×; 400×. (**e**) The collagen fibre (staining green) and myofibre (staining red) of bile duct anastomosis in the AgNPs group and control group were observed using Masson’s trichrome staining. Magnification: 400×.

To monitor the biliary status in real time, blood samples were collected serially via an ear vein at 4, 12, 24 and 48 weeks after biliary stent implantation to test liver function in the other 24 pigs in each group. We found that plasma total bilirubin (TBIL) and direct bilirubin (DBIL) persistently increased from 12 to 24 to 48 weeks post-operation, and plasma γ-L-glutamyl dipeptide (γ-GT) presented a continuous increase from 4 to 48 weeks post-operation in the control group (Fig. 6a–c). Importantly, the application of the AgNP biliary stent significantly decreased the levels of plasma TBIL and DBIL versus the control from 12 to 24 to 48 weeks post-operation (all p < 0.01 or 0.001, Fig. 6a,b); additionally, the plasma γ-GT level increased from 4 to 48 weeks post-operation (all p < 0.001, Fig. 6c). These results indicated that the application of the AgNP biliary stent maintained an unobstructed biliary system and maintained good biliary status *in vivo*.

To further investigate bacterial infection in bile ducts, six pigs in each group at each time point were sampled and their bile was collected for bacterial culture. We found that the positive rate of bacterial culture in the bile was persistently elevated in the control group from 4 to 12 to 24 to 48 weeks post-operation, corresponding to 0%, 33.33%, 50% and 100%, respectively. Notably, the bacterial culture was consistently negative in the AgNPs group from 4 to 48 weeks post-operation (Table 1). Furthermore, the selected bacterial culture media were used to identify positive bacteria in the bile. The results indicated that the positive bacteria in the bile were primarily *Escherichia coli*, *Staphylococcus aureus*, *Quail chicken enterococcus D*, *Enterobacter cloacae*, *Klebsiella pneumoniae* and *Enterococcus faecalis*, of which *Escherichia coli* was the dominant bacteria, accounting for 6/11 (54.55%) (Table 2). These data suggested that the application of the AgNP biliary stent could significantly decrease bacterial infection in the bile duct and maintain long-term unobstructed status *in vivo*.

In addition, the anastomosis of the bile duct was evaluated to perform H&E and Masson’s trichrome staining at 48 weeks post-operation. The scar tissue was significantly formed, and substantial fibrocytes could be observed in the control group, whereas the degree and scope of fibrosis were attenuated in the AgNP group, as shown histologically (Fig. 6d). Notably, the collagen fibre (staining green) was significantly more abundant, whereas the myofibre (staining red) was remarkably decreased, in the control group versus the AgNP group (Fig. 6e). These results suggested that the application of the AgNPs biliary stent significantly decreased the formation of collagen fibres and scar tissue on the anastomosis of bile ducts *in vivo*.

## Discussion

Symptomatic biliary strictures can lead to various adverse outcomes, including chronic cholestasis, jaundice, recurrent cholangitis and secondary biliary cirrhosis, and these complications can be serious, irreversible, and life threatening^1^. Fully covered self-expanding metal stents (FCSEMSs) are gaining acceptance for the treatment of benign biliary strictures and palliative management of malignant biliary obstructions^1^,^8^,^28^,^29^. FCSEMSs have been clinically applied in the treatment of benign biliary strictures, including biliary fistula post-operation, primary sclerosing cholangitis^2^, pancreatitis^3^ and biliary complications after liver transplantation^5^, as well as in the management of malignant biliary obstructions. However, the long-term unobstructed rate of the FCSEMSs clinically remains limited. Thus, development of a novel biliary stent is essential for clinical applications. In this study, we developed a novel biliary stent coated with AgNPs and investigated its function *in vitro* and *in vivo*. First, we identified the characteristics and functional properties of the AgNP biliary stent *in vitro* and found that the AgNP biliary stent possessed a long-term anti-coagulation effect and a high-efficiency anti-bacterial function for both short- and long-term periods. We also noted that the Ag^+^ release in the AgNP biliary stent was an effective and slow process. Importantly, the application of the AgNP biliary stent significantly prolonged the unobstructed period of the biliary system and improved survival time in the preclinical study. These advantages may be attributed to the effective anti-microbial function and the decreased granular tissue formation on the surface of the anastomotic biliary. These findings may provide a novel and effective strategy for the treatment of symptomatic biliary strictures.

Nanoparticles have become a well-recognized, effective antimicrobial substance^16^,^17^ because of their high surface-to-volume ratio. AgNPs show a wide range of antibacterial characteristics as well as virucidal effects and thus are used in many disinfectant agents and medical devices^18^,^30^,^31^. AgNPs can adhere to the cell walls of bacteria and penetrate the cell, resulting in increased permeability and disintegration of the bacterial cell membrane^16^,^32^. In our *in vitro* study, we first identified the characteristics of the AgNP complex using ultraviolet detection and found that the obtained AgNP complex was stable without AgNP agglomeration and that the abundance of Ag in the AgNP complex correspondingly increased with an increase in the bilayer number. Topographical observation of the AgNP biliary stent indicated that the spin-assembly method presented high quality and efficacy. We also noted that the AgNP biliary stent exhibited a long-term anti-coagulation effect. Interestingly, after storage of the AgNP biliary stent for 1 month, the survival ratio of *E. coli* continuously declined with the co-culture of AgNPs and *E. coli*, which suggested that the AgNPs biliary stent possessed a sustained high-efficiency anti-bacterial function. This function exists because the Ag^+^ release in the AgNP biliary stent is an effective and slow process. Because of its high-efficiency anti-bacterial function for both short- and long-term periods, the AgNP complex has promising potential for application.

Because of biliary re-strictures or obstruction, the sequential placement of plastic stents in increasing numbers over a 1-year period, requiring exchanges approximately every 3 months, has been the established endoscopic approach for the management of symptomatic biliary strictures^11^. Despite the high success rates, this approach is technically demanding and requires an average of 5 incidences of endoscopic retrograde cholangiopancreatography (ERCP)^33^. Multiple interventions for necessary stent exchanges are also burdensome to patients; thus, a better approach is to develop a novel biliary stent with a longer unobstructed period of the biliary system. Bacterial infection is the leading factor of bacterial biofilm formation^13^,^14^, resulting in biliary stent re-strictures^12^,^13^. Therefore, we developed a novel biliary stent coated with AgNPs and performed a preclinical study in sixty-four pigs. From each group, eight pigs were randomly selected in which to observe the percentage of biliary obstruction and survival function.

Our results indicated that the application of the AgNP biliary stent significantly delayed the occurrence of biliary obstruction versus the control. Correspondingly, the Kaplan-Meier survival curve demonstrated that the survival function was remarkably increased in eight pigs from the AgNP group versus the control. These data indicated that the application of the AgNP biliary stent significantly prolonged the unobstructed period of the bile duct and improved survival time in the preclinical study. The other forty-eight pigs were used to dynamically investigate liver function and bacterial infection in the bile at different time points in both groups. The results indicated that application of the AgNP biliary stent can maintain good liver function status and significantly decrease bacterial infection in the biliary system *in vivo*. We also noted that the application of the AgNPs biliary stent significantly decreased the formation of collagen fibres and scar tissue on the anastomosis of the bile duct *in vivo*. Together, these factors contributed to a long-term unobstructed status of the biliary system after the application of the AgNPs biliary stent.

Bacterial infection is the leading factor of bacterial biofilm formation^13^,^14^, resulting in biliary stent re-strictures^12^,^13^. Because of a high incidence of bacterial infection after biliary injury^27^,^34^, bacterial adherence and biliary sludge are prone to occur, inducing biliary stent occlusion. Some studies have indicated that the formation of bacterial biofilm is the main cause for biliary stent re-strictures^12^,^13^. Notably, bacteria is the leading factor of bacterial biofilm formation^13^,^14^. In patients with biliary stents, a wide range of different branches and groups of bacteria participate in the development of biofilms on the surfaces of biliary stents, and the occlusion of stents leads to progressive extinction of the biofilm and mummification of its components. Finally, the mummification of the biofilm and the deposition of cholesterol or other substances within the biofilm matrix aggregate to develop biliary sludge and then block biliary stents^35^,^36^. In patients with biliary strictures caused by acute cholangitis, the predominant strains in bile or blood culture were the *Enterococcus* species, followed by *Escherichia coli* and *Klebsiella* species^34^. The incidences of the *Enterococcus* species and non-fermenters were significantly increased in cholangitis episodes with versus without a biliary stent. Particularly, more *Pseudomonas aeruginosa* and *Enterococcus faecium* were observed in patients with a biliary stent^34^. However, in a porcine model with a biliary stent, we found that the positive bacterial rate in bile was persistently elevated in the control group, whereas the bacterial culture was consistently negative in the AgNP group from 4 to 48 weeks post-operation. Additionally, the positive bacteria in bile were primarily *Escherichia coli*, *Staphylococcus aureus*, *Quail chicken enterococcus D*, *Enterobacter cloacae*, *Klebsiella pneumoniae* and *Enterococcus faecalis*; *Escherichia coli* was the dominant bacteria. Additionally, our *in vitro* experiment indicated that the AgNP biliary stent exhibited a high-efficiency anti-bacterial function for both short- and long-term periods and resisted various bacteria, including *E. coli, Staph. aureus*, *Enterococcus* and *P. aeruginosa*. Thus, our AgNP biliary stent possesses a considerable advantage and can resist bacterial infection *in vivo* and *in vitro*, significantly contributing to the long-term unobstructed status of the biliary system.

FCSEMSs have been recognized as an optimal therapy and have been widely used in clinics for benign biliary strictures and palliative management of malignant biliary obstructions. In our preliminary experiment, both naked metal stents and fully covered self-expanding metal stents were used to study the efficacy both *in vitro* and *in vivo* of AgNP complex stents. However, the results from our preliminary experiment indicated that it is very difficult to coat a AgNP complex on both the external layer and the inner layer of metal stents. The AgNP complex can be coated only if other materials were previously coated on the surface of the metal stent, which may interfere with the efficacy of the AgNP complex and experimental results. In contrast, the metal stent was compressed in a pushing system with the inner and outer sheath *in vitro*, and it has a relative length and hardness. If the metal stent was removed from the inner and outer sheaths of the pushing system, the metal stent would expand and the diameter of the metal stent would become greater than that of the common bile duct. Thus, the metal stent cannot be placed in the common bile duct of pigs. Therefore, in our formal experiment, metal stents were abandoned and we selected plastic stents as the study subject.

In conclusion, we developed a novel biliary stent coated with AgNPs. The AgNP biliary stent exhibited a high-efficiency anti-bacterial function for both short- and long-term periods, which may be attributed to the slow release of Ag^+^ in the AgNP biliary stent. Importantly, the application of the AgNP biliary stent significantly prolonged the unobstructed period of the biliary system and improved survival time in the preclinical study. These advantages may be attributed to the effective anti-microbial function and decreased granular tissue formation on the surface of the anastomotic biliary. These findings may provide a novel and effective strategy for the treatment of symptomatic biliary strictures.

## Additional Information

**How to cite this article**: Yang, F. *et al.* A novel biliary stent coated with silver nanoparticles prolongs the unobstructed period and survival via anti-bacterial activity. *Sci. Rep.* **6**, 21714; doi: 10.1038/srep21714 (2016).

## Figures and Tables

**Table 1:** The positive rate of bacterial culture in bile from different groups at different time points.

Groups/time	Post 4w	Post 12w	Post 24w	Post 48w
Control	0/6 (0)	2/6 (33.33%)	3/6 (50%)	6/6 (100%)
AgNPs	0/6 (0)	0/6 (0)	0/6 (0)	0/6 (0)
P value	1.00	0.4545	0.1818	0.0022

Note: Fisher’s exact test was used for statistical analysis.

**Table 2:** Identification of bacterial culture in the bacteria-positive bile samples.

Bacterial identification	Occurrence rate
*Escherichia coli*	6/11 (54.55%)
*Staphylococcus aureus*	1/11 (9.09%)
*Quail chicken enterococcus D*	1/11 (9.09%)
*Enterobacter cloacae*	1/11 (9.09%)
*Klebsiella pneumoniae*	1/11 (9.09%)
*Enterococcus faecalis*	1/11 (9.09%)
